# A Mixed Model of Clinical Characteristics, Strain Elastography and ACR-TIRADS Predicts Malignancy in Small Thyroid Nodules: A Prospective Single-Center Study

**DOI:** 10.3390/medicina61101774

**Published:** 2025-10-01

**Authors:** Nikolaos Angelopoulos, Emmanouil Petropoulos, Ioannis Chrisogonidis, Sarantis Livadas, Rodis D. Paparodis, Ioannis Androulakis, Juan Carlos Jaume, Dimitrios G. Goulis, Ioannis Iakovou

**Affiliations:** 1 2nd Academic Department of Nuclear Medicine, Faculty of Medicine, School of Health Sciences, AHEPA University Hospital, 54636 Thessaloniki, Greece; drangelnick@gmail.com (N.A.); iakovou@otenet.gr (I.I.); 2 Hellenic Endocrine Network, 6, Ermou St., 10563 Athens, Greece; rodis@paparodis.gr (R.D.P.); i-androulakis@hen.gr (I.A.); 3 Center of Fine Needle Aspiration, Vasilissis Sophias 124B St., 11526 Athens, Greece; info@petropoulos.com; 4 Department of Radiology, Faculty of Medicine, School of Health Sciences, Aristotle University of Thessaloniki, 54124 Thessaloniki, Greece; chryssog@med.auth.gr; 5 Endocrine Unit, Athens Medical Centre, 11525 Athens, Greece; 6 Department of Medicine, Division of Endocrinology, Diabetes and Metabolism, Loyola University Chicago/Edward Hines Jr. VA Hospital, Hines, IL 60141, USA; juan.jaume@va.gov; 7 Unit of Reproductive Endocrinology, 1st Department of Obstetrics—Gynecology, Medical School, Aristotle University of Thessaloniki, 54124 Thessaloniki, Greece; dgg@auth.gr

**Keywords:** thyroid nodules, malignancy, ultrasound, real-time elastography

## Abstract

*Background and Objectives*: To identify clinical, ultrasound (US) and real-time elastography (RTE) characteristics indicative of malignancy in small thyroid lesions. *Materials and Methods*: 141 consecutive patients with incidentally discovered solid thyroid nodules (diameter ≤ 10 mm) by neck US were assessed, and RTE was performed. The nodules were classified per American (ACR-TIRADS) and European (EU-TIRADS) criteria; US-guided FNA was conducted on EU-TIRADS 5 nodules. The US and RTE features of nodules classified as benign (Bethesda II) or malignant (Bethesda V and VI) were compared. *Results*: 41 nodules were classified as EU-TIRADS 5. Their Fine Needle Aspiration (FNA) cytology was Bethesda II (*n* = 11), III-IV (*n* = 3), V (*n* = 10) or VI (*n* = 17). Bethesda V–VI patients had a higher rate of autoimmune thyroiditis (*p* = 0.015) and higher ACR-scoring points (*p* < 0.001) compared with Bethesda II. The elastography ratio was equal between the groups (*p* = 0.584). In logistic regression analysis, ACR-scoring points predicted FNA results, with an area under the curve (AUC) of 0.993 (sensitivity 92.6% and specificity of 100%). The clinical model (age, body mass index, sex, autoimmunity, L-thyroxine treatment, nodule diameter, elastography ratio) achieved an AUC of 0.744. A “mixed” model, combining clinical characteristics with the ACR scoring points, achieved perfect performance (AUC = 1.000), predicting FNA results with 100% sensitivity and specificity. *Conclusions*: The proposed “mixed model” can predict Bethesda V–VI in thyroid nodules <10 mm, allowing for the selection of those needing further evaluation.

## 1. Introduction

The availability of point-of-care ultrasonography (US), high-resolution US and US-guided fine needle aspiration (FNA) biopsy has facilitated the identification and the cytological diagnosis of nodules as small as 3 mm in diameter [1]. This fact is partly responsible for the increased incidence of papillary thyroid carcinoma (PTC) [2]. Because most PTCs with a diameter ≤10 mm do not cause clinical disease [3], the American Thyroid Association (ATA) advises against FNA of thyroid nodules ≤10 mm unless they exhibit suspicious US features (marked hypoechogenicity, irregular margins, punctate echogenic foci, taller-than-wide shape) [1,4]. Nevertheless, other societies, such as the Japanese Association of Endocrine Surgery, recommend early FNA to stage and manage such nodules [5]. The increasing detection of sub centimeter thyroid nodules—largely due to incidental findings on imaging—has raised concerns about potential overdiagnosis and overtreatment [6]. Many of these nodules are clinically indolent, and the challenge lies in differentiating which small lesions harbor malignancy and warrant further intervention [7]. This clinical dilemma is compounded by inconsistencies between international guidelines regarding the indications for FNA in nodules ≤10 mm [8].

Various risk stratification systems, such as the Thyroid Imaging Reporting and Data System (TIRADS), use US features to assess the risk of malignancy of thyroid nodules and guide the decision for further FNA evaluation [9]. Several studies [10,11,12] demonstrated that adding real-time elastography (RTE) to B-mode and color-Doppler US increases the sensitivity for detecting thyroid malignancy. However, the diagnostic accuracy depends on the nodule size, with reduced accuracy in nodules ≤10 mm compared with those >10 mm [13]. In particular, the performance of both conventional ultrasonographic scoring systems (e.g., ACR-TIRADS) and elastographic methods appear to decline in small nodules [14], where size alone may limit interpretability. Furthermore, TIRADS does not account for clinical parameters such as autoimmune thyroiditis, patient sex, body mass index (BMI), or levothyroxine treatment—factors that may independently influence malignancy risk.

Thus, there is a growing interest in developing models that combine imaging and clinical data to improve risk stratification [15]. Therefore, there is a need to develop tools to discriminate those patients at risk and manage them accordingly. The present study aimed to fill this gap by creating a “mixed” predictive model that integrates clinical variables (age, sex, BMI, thyroid autoimmunity, levothyroxine use) with US and RTE features to assess malignancy risk in small (≤10 mm), suspicious thyroid nodules.

## 2. Materials and Methods

### 2.1. Patients

Adult individuals presenting with incidentally discovered small (≤10 mm) solid thyroid nodules who visited an outpatient clinic in Greece between June 2023 and June 2024 were enrolled. Inclusion criteria were age ≥ 18 years, presence of at least one solid thyroid nodule with a maximum diameter ≤10 mm on ultrasonography, and provision of written informed consent. Exclusion criteria included: previous thyroid surgery, history of neck irradiation, known or suspected familial thyroid cancer syndromes, current or prior diagnosis of thyroid malignancy, inability to tolerate or undergo ultrasound or FNA procedures, and indeterminate cytology (Bethesda I, III, or IV). Patients receiving active surveillance or treatment for any malignant condition were also excluded.

Those consenting to participate were evaluated with a real-time two-dimensional grey scale and Doppler US by the same experienced endocrinologist. Data on age, sex, height, and weight were collected, and body mass index (BMI) was calculated as weight (kg)/height^2^ (m^2^). The history of hypothyroidism and use of L-thyroxine were recorded. Autoimmune thyroid disease (AT) was documented by the presence of anti-peroxidase (anti-TPO) and/or anti-thyroglobulin (anti-Tg) antibodies. Information regarding other comorbidities (e.g., metabolic syndrome, diabetes) was collected but not used in the primary analysis. Smoking status and family history of thyroid disease were not recorded. The number of thyroid nodules in each subject was recorded, along with their US characteristics, such as size (anteroposterior, transverse, longitudinal), shape (wider-than-tall or taller-than-wide), composition (spongiform, solid, cystic, mixed), echogenicity (hyperechoic, isoechoic, hypoechoic, markedly hypoechoic), margins (smooth, ill-defined, lobulated, irregular, extrathyroidal extension), and calcifications/punctate echogenic foci (comet-tail artifacts or indeterminate, peripheral calcifications, macrocalcifications, microcalcifications).

All measurements were obtained by a single operator, and the classification of sonographic features was reviewed independently by two additional endocrinologists. American (ACR-TIRADS) and European (EU-TIRADS) scorings were calculated for each nodule by two qualified endocrinologists in an independent and blinded fashion (RP, SL). A third endocrinologist (NA) decided the scores when disagreement occurred. The agreement between the two raters was assessed using Cohen’s kappa coefficient. All nodules were stratified to the five TIRADS categories [TR1 (0 points): benign; TR2 (2 points): not suspicious; TR3 (3 points): mildly suspicious; TR4 (4–6 points): moderately suspicious; TR5 (≥7 points): highly suspicious] [16].

### 2.2. Methods

A General Electric P9 device (LOGIQ-P9^®^; GE Medical Systems, Milwaukee, WI, USA) with an 8–12 MHz multifrequency linear probe was used for conventional B-mode thyroid US. All sonographic examinations were performed by the same experienced endocrinologist with over 10 years of clinical imaging experience, using standardized settings for depth, gain, and focus. The evaluation included both transverse and longitudinal views, with special attention to the reproducibility of measurements.

Real-time elastography (RTE) was conducted using the same ultrasound equipment [17]. In brief, mild external pressure was applied to obtain valid images, and the interpretation was made using a red-green-blue color map: soft tissue is indicated by red, intermediate stiffness by green (equal strain), and hard nodules by blue. To ensure measurement reliability, each nodule was scanned at least twice, and the average value of the strain ratio (SR) was recorded. To semi-quantitatively determine the strain ratio (SR), a numeric value was obtained by comparing two regions of interest (ROI) placed at the same depth: the strain value of the proximal sternocleidomastoid muscle (E1) and the mean strain of the nodule (E2), with SR = E_2_/E_1_. Transverse sections were used for consistency, and care was taken to include both the nodule and reference muscle within the same image frame. Measurements were discarded and repeated if motion artifacts or improper compression were observed.

US-guided FNA was performed only in nodules classified as EU-TIRADS 5. The procedure was carried out using a 23 G needle and standard aspiration technique without local anesthesia. The cytological samples were immediately air-dried and alcohol-fixed for evaluation by an experienced cytopathologist blinded to the clinical and imaging findings. The cytological evaluation was performed at the Aristotle University Cytology Laboratory in Thessaloniki, Greece.

Nodules were classified according to the Bethesda system as benign (Bethesda II) or malignant (Bethesda V–VI). Patients with Bethesda I, III, or IV cytology results were excluded from the final analysis to ensure clear diagnostic classification. No patient with Bethesda II underwent surgery during follow-up, so histopathologic confirmation was unavailable for benign nodules.

### 2.3. Statistical Analysis

Continuous variables were reported as mean and standard deviations, while categorical variables were reported as frequency and percentage. Normality was assessed using the Shapiro–Wilk test. Comparisons between groups were performed using the Mann–Whitney U test for continuous variables and the chi-squared test for categorical variables.

Logistic regression analysis was performed to examine the predictive power of independent variables to discriminate thyroid malignancy. A univariate logistic regression was initially conducted for each variable of interest. Variables with *p* < 0.10 were entered into multivariate logistic regression models. Multicollinearity was evaluated using the variance inflation factor (VIF), and all included variables had VIF < 2, indicating acceptable independence.

Three models were constructed: (1) an “ACR model” based solely on the ACR-scoring points; (2) a “clinical model” including clinical and demographic variables (age, sex, BMI, autoimmunity, L-thyroxine use, nodule diameter, and elastography ratio); and (3) a “mixed model” integrating both the clinical parameters and the ACR scoring points. All models were created using the enter method (forced entry).

The Receiver Operating Characteristic (ROC) analysis was used to evaluate the predictive performance of the models for Bethesda V–VI classification. The area under the curve (AUC), Youden Index, sensitivity, and specificity were calculated from the ROC analysis. Pairwise comparison of AUCs between the three models was performed using the DeLong test. Confidence intervals for AUCs were computed at the 95% level. Statistical analyses were conducted using MedCalc^®^ software version 19.5.3 (MedCalc^®^ Software, Ostend, Belgium). A *p*-value < 0.05 was considered statistically significant. There were no missing data in the dataset; all variables were complete for all included patients.

## 3. Results

**Figure 1:**
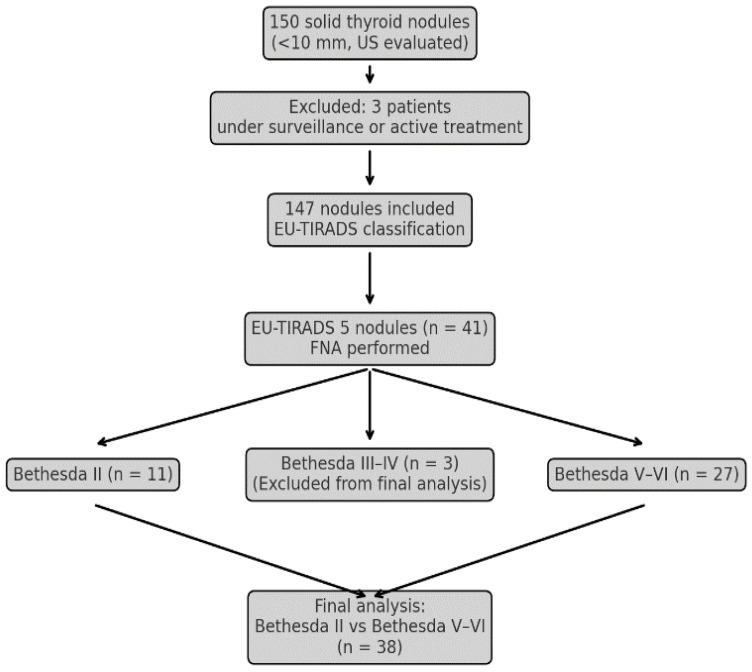
Flowchart of patient selection and nodule classification.

Of 147 consecutive solid thyroid subcentimeter (range 4.7–9.8 mm) nodules, 41 were classified as EU-TIRADS 5 [the remaining were classified as 4 (*n* = 67), 3 (*n* = 38) or 2 (*n* = 1)] and underwent FNA. The ACR-TIRADS scores were independently evaluated by two experienced endocrinologists, with concordance in 96.6% of cases (142 out of 147 nodules). The calculated Cohen’s kappa coefficient (κ = 0.93) demonstrated almost perfect interobserver agreement. According to the FNAs results, nodules classification was Bethesda II (*n* = 11), III (*n* = 2), IV (*n* = 1), V (*n* = 10) or VI (*n* = 17). The final study population consisted of 38 patients (Table 1 and Figure 1).

**Figure 2:**
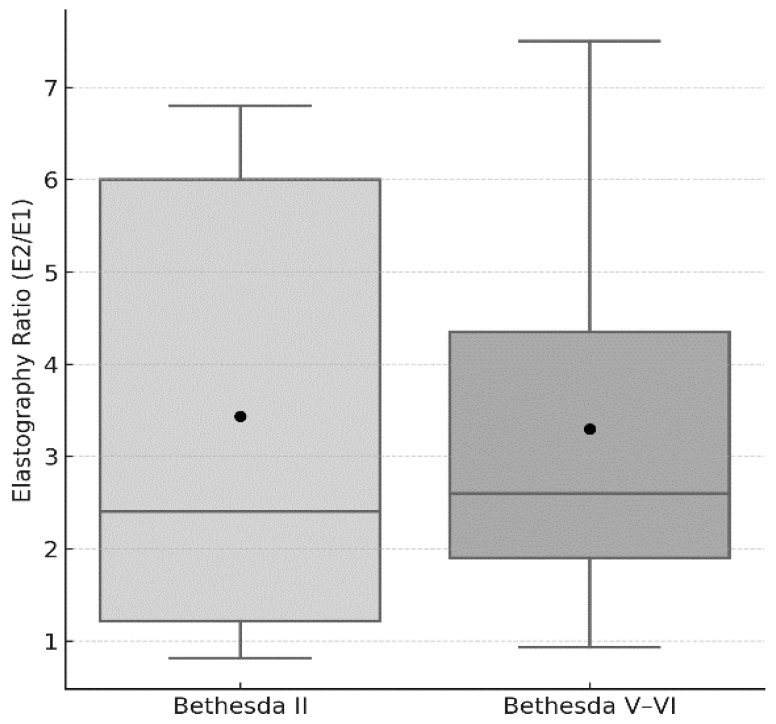
Comparison of elastography ratios (E_2_/E_1_) between cytologically benign and malignant thyroid nodules.

A classification of Bethesda V–VI was more frequent in individuals with autoimmune thyroiditis (Chi-squared 5.833, *p* = 0.015) compared with those with Bethesda II. No other difference was detected between the groups in clinical/demographic parameters (Table 1). The elastography ratio (E_2_/E_1_) was similar between the groups (3.3 ± 1.9 Vs. 3.2 ± 2.3, *p* = 0.584) (Figure 2). In Bethesda V–VI, ACR-scoring points and ACR-TIRADS categories were higher compared with Bethesda II.

Box-and-whisker plot comparing the elastography ratio (E_2_/E_1_) between cytologically benign (Bethesda II) and malignant (Bethesda V–VI) thyroid nodules. Boxes indicate the interquartile range (25th–75th percentile), the horizontal line within each box marks the median, whiskers represent the data range within 1.5 × IQR, and black circles denote the mean values. No statistical outliers were detected in either group. Comparison by Mann–Whitney U test: *p* = 0.584.

### 3.1. Logistic Model

In a logistic regression analysis, ACR-scoring points (>6) predicted the Bethesda V–VI classification (Table 2). The ROC analysis (AUC 0.993, Youden Index 0.926) had a sensitivity of 92.6% and a specificity of 100%.

### 3.2. Clinical Model

To analyze the predictive power of the clinical parameters for Bethesda V–VI classification, clinical parameters (age, BMI, sex, autoimmunity, L-thyroxine supplementation, nodule largest diameter) and elastography ratio were examined in a “clinical” model. Its predictive value was significant (*p* = 0.003, AUC 0.744, Youden Index 0.505) with a sensitivity of 77.8% and specificity of 72.7%.

### 3.3. Mixed Model

**Figure 3:**
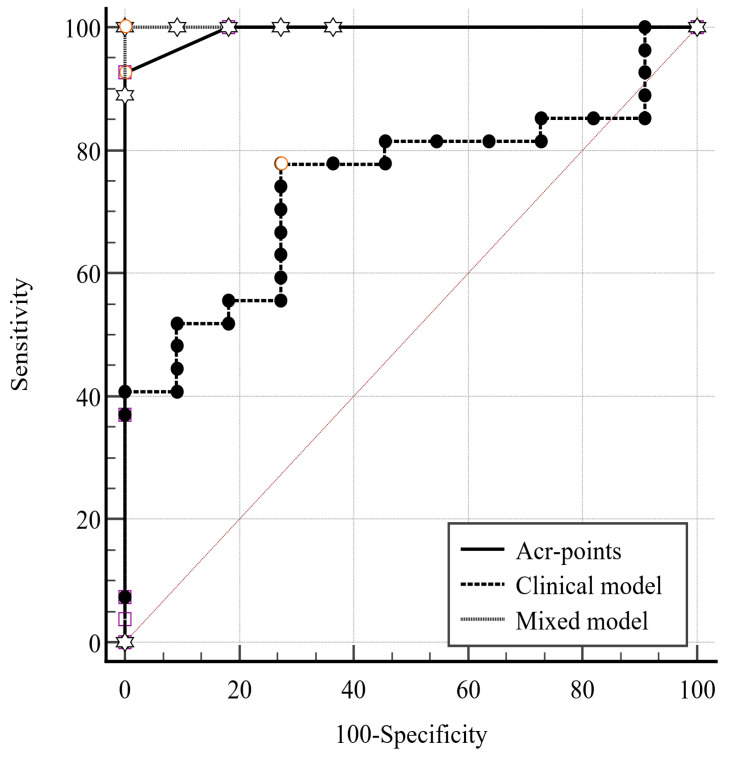
ROC curves of our predictive models. ACR: American College of Radiology; ROC: receiver operating characteristic.

Finally, a “mixed” model was generated, combining the clinical characteristics mentioned above with the addition of the ACR scoring points. The mixed model was significant (*p* < 0.001, AUC 1.0, Youden Index:1) with a sensitivity and specificity of 100%. A pairwise comparison of the three models is illustrated in Table 3 and Figure 3.

## 4. Discussion

The prevalence of thyroid nodular disease increases with age, surpassing 50% in those >65 years [18,19]. To enhance nodule assessment, risk stratification systems (RSS) have been developed to determine malignancy risk and assign score points for specific US features. The EU-TIRADS and the ACR-TIRADS have high predictive values and assist in selecting nodules that warrant further assessment with FNA [20]. An important feature of all RSS is using a size threshold to determine the need for FNA, which differs for specific subgroups. In nodules at the highest malignancy risk (TIRADS 5), all RSS mandate an FNA when they exceed 10 mm in largest diameter. No guideline recommends FNA in nodules ≤10 mm, independently of other characteristics.

The guidelines for performing FNA in small thyroid nodules have several discrepancies among scientific societies [e.g., American Thyroid Association (ATA), European Thyroid Association (ETA)] regarding nodule size and characteristics, risk stratification, patient factors, surveillance, and cost-effectiveness [21].

This heterogeneity reflects a deeper uncertainty about the biological behavior and clinical relevance of subcentimeter thyroid nodules. Some data suggest that a significant number of papillary microcarcinomas (≤10 mm) follow an indolent course [22], while others highlight cases with lymph node metastases or aggressive features, even at small sizes [23,24]. These discrepancies highlight the need for tools that can differentiate patients at high risk, even when nodules are small.

The discrepancies mentioned above are based on the fact that the clinical significance of detecting malignancy in small thyroid nodules is debatable [3,25]. Many differentiated thyroid malignancies detected in small nodules have an indolent course, grow slowly, and are less likely to affect mortality [22]. Detecting small, low-risk thyroid malignancies may lead to overtreatment, exposing patients to unnecessary procedures and potential complications without long-term benefits [26]. On the other hand, early diagnosis of a malignant nodule, while small in size, might ease follow-up and guide clinical decisions [23,24].

Our study addresses this clinical gap by providing a predictive framework that integrates both imaging-based risk scores and individual patient characteristics. The mixed model we propose combines ACR-TIRADS scoring with age, BMI, sex, autoimmune thyroid disease, levothyroxine use, and elastography findings to achieve high diagnostic performance. The limited data available for conventional ultrasound performance makes it challenging to draw comprehensive comparisons with strain elastography. However, the reported sensitivity and specificity values for conventional ultrasound appear generally lower than those reported for strain elastography in the same studies. Some studies [27,28] reported very high sensitivity (100%), while others [29] reported lower values. This variability could be due to differences in study populations, elastography techniques, or scoring systems. Only a few studies [11,30,31] explicitly mentioned focusing on nodules ≤10 mm and reported strain elastography sensitivities of approximately 90.6% and 88.9% with corresponding specificities near 89%.

A single study reporting combined modality data [32] showed high sensitivity (97.1%) and NPV (91.9%), suggesting potential benefits of combining strain elastography with conventional ultrasound. In the present study, US and RTE features provided data for a “mixed” model to predict the Bethesda V–VI classification (as a proxy to malignancy risk) in small yet suspicious (EU-TIRADS 5) thyroid nodules. Our logistic regression models demonstrated that while ACR-TIRADS scoring alone had high accuracy (AUC 0.99), combining it with selected clinical factors yielded perfect sensitivity and specificity (AUC 1.00). This suggests that clinical context adds valuable predictive power, especially in ambiguous or borderline cases. Focusing on the subgroup of patients with specific ultrasound findings suspicious of malignancy (EU-TIRADS 5), we found that the prevalence of Bethesda V and VI was 61%, which follows the high reported risk in the literature (26–87%) [33]. These findings are consistent with and extend prior studies exploring the diagnostic value of US and elastography in small thyroid nodules. For example, Dighe et al. [27] examined nodules ≤ 10 mm and reported that RTE alone achieved a sensitivity of 88.9% and specificity of 89%, while conventional ultrasound alone performed less effectively. Similarly, Wang et al. [29] noted that RTE improved diagnostic performance, but still showed variability depending on operator and technique.

In contrast to those studies which evaluated elastography or TIRADS in isolation, our study combined both approaches with relevant clinical data to produce a unified predictive model. A recent multicenter study by Hairu et al. [32] used combined US and elastography but did not incorporate patient-specific parameters such as age or BMI. Their reported sensitivity (97.1%) and negative predictive value (91.9%) were comparable to our “mixed model,” but our approach reached 100% sensitivity and specificity within a focused high-risk group.

Moreover, unlike earlier works that included nodules of varying sizes, our study strictly focused on subcentimeter nodules classified as EU-TIRADS 5—a population for which there is limited but clinically critical evidence. Prior studies such as that of Trimboli et al. [11] suggested that elastography could increase diagnostic accuracy, yet their cohorts included nodules up to 20 mm. Our model not only confirms that finding but also refines it by demonstrating that high performance can be maintained even in the most challenging subgroup: nodules ≤ 10 mm.

Implementing such a model in daily practice could help identify nodules that are likely to be malignant and require appropriate intervention, compared to benign ones. Such a strategy would reduce the use of resources (radiologists’ and pathologists’ time/cost, equipment use, healthcare system costs) in following up on nodules that are unlikely to produce harm. On the other hand, early detection of malignant thyroid nodules can lead to better care strategies, even if they include active surveillance.

Importantly, our model could be particularly helpful in decision-making for borderline nodules—those with ACR scores of 5–6, where FNA is not typically recommended. The ability to integrate clinical red flags (e.g., male sex, autoimmunity, elevated BMI) could shift the threshold toward early evaluation or closer follow-up in selected individuals. In addition, our investigation sought to assess the impact of additional clinical variables on the malignant cytology rates within the ACR TI-RADS categories, including age, BMI, thyroxine use, and autoimmune thyroiditis history. In both classification systems, nodule stratification is exclusively based on US characteristics without regard to clinical variables. The potential for malignancy is based on a variety of clinical variables such as younger age, male sex, and nodule size, allowing for an “individualization” of thyroid nodule management [34].

The risk of malignant thyroid nodules decreases as age increases, while the prevalence of such nodules increases [35]. TIRADS has a low specificity (28%) in groups of patients, such as those >70 years, and the number of superfluous biopsies has increased [36]. An elevated BMI has been positively associated with thyroid cancer risk, particularly in women [37]. This fact may be the result of factors (insulin resistance, obesity-related inflammation, hormonal alterations), which are more prevalent in individuals with a higher BMI [38,39].

While some studies have associated thyroid cancer risk in patients with primary hypothyroidism [40], others have not [41]. High thyroid peroxidase antibody (anti-TPO) titers have been associated with a reduced risk of thyroid malignancy in patients with Hashimoto’s thyroiditis [42]. The significance of chronic lymphocytic thyroiditis as a risk factor for differentiated thyroid carcinoma is a matter of debate.

Strengths of our study include the prospective design, blinded and independent US/TIRADS scoring by multiple endocrinologists, and high interobserver agreement. The use of a well-defined and narrowly focused population (only EU-TIRADS 5 nodules ≤10 mm) allows for direct clinical applicability.

This study also has limitations. First, no histopathological confirmation was available for Bethesda II nodules, potentially underestimating the true malignancy rate. Second, due to the high-risk selection (EU-TIRADS 5 only), the model may not generalize to nodules in lower-risk categories. Third, the small sample size—though typical for this type of study—may limit the external validity and precision of the estimates. Larger multicenter studies are warranted to confirm these findings.

Although serum TSH was not included in our proposed predictive models, it is acknowledged as a potentially valuable parameter in thyroid nodule risk stratification. A recent study by Trimboli et al. [43] demonstrated that incorporating TSH values into existing TIRADS classifications significantly enhanced diagnostic sensitivity for detecting differentiated thyroid cancer (DTC) (AUC = 0.70 for TSH > 1.3 mIU/L). Notably, TSH remained an independent predictor of malignancy alongside TIRADS score and patient gender in multivariate analysis. The combination of TIRADS and TSH increased the sensitivity of ACR-TIRADS from 78.9% to 92.1%, though at the cost of higher unnecessary FNAC rates. These findings are in line with prior evidence suggesting an association between higher TSH levels and increased risk of DTC, especially in patients with indeterminate cytology. Cappelli et al. [44], demonstrated that even among euthyroid patients with thyroid nodules of indeterminate cytology, higher serum TSH was independently associated with increased malignancy risk. These findings suggest that the integration of TSH into diagnostic algorithms—particularly in ambiguous or borderline cases—may enhance risk stratification and guide the decision-making process in clinical practice.

In our study, TSH values were not systematically available for all participants and were therefore not included in the multivariable analysis. We recognize this as a limitation and plan to assess the potential contribution of TSH in future models. Given its wide availability and low cost, the integration of TSH into clinical or mixed predictive models may offer additional diagnostic value, especially in borderline or uncertain cases. Moreover, regarding clinical outcomes, nodules classified as Bethesda II are associated with a very low malignancy risk (<3%) and are typically managed conservatively without surgery, unless clinical or sonographic suspicion arises [45].

On the other hand, Bethesda III (Atypia of Undetermined Significance/Follicular Lesion of Undetermined Significance, AUS/FLUS) cytology poses a diagnostic challenge due to its intermediate malignancy risk, estimated between 6% and 30% depending on institutional thresholds and ancillary tools such as molecular testing or repeat FNA [46]. Surgical management is often considered, especially in cases with suspicious ultrasound features or persistent indeterminate cytology. Recent meta-analyses have highlighted significant variability in outcomes across centers, with malignancy rates in resected Bethesda III nodules ranging from 10% to 30% [47]. As reported by Liu et al. [48], in a cohort of 188 surgically treated AUS/FLUS nodules, the malignancy rate reached 54.3%, and multivariate analysis identified male sex, a taller-than-wide shape, microcalcifications, and the presence of the BRAF V600E mutation as independent predictors of thyroid cancer. This variability underscores the need for individualized risk stratification and further supports the role of multimodal prediction models in improving clinical decision-making.

## 5. Conclusions

In conclusion, we conducted FNA evaluations in individuals with small (≤10 mm) thyroid nodules exhibiting suspicious US characteristics (EU-TIRADS 5). According to the current guidelines, FNA is not recommended for these nodules. The proposed “mixed” model of clinical, US and RTE features can predict Bethesda V–VI classification in small nodules, allowing for selection of those needing further evaluation.

## Figures and Tables

**Table 1:** Descriptive statistics of EU-TIRADS 5 nodules.

	Bethesda II (*n* = 11)	Bethesda V–VI (*n* = 27)	*p*-Value
**Age** (years)	45.0 ± 13.6	47.3 ± 13.3	ns
**BMI** (kg/m^2^)	25.7 ± 4.4	26.2 ± 5.4	ns
**Diameter** (mm)	7.6 ± 2.0	7.8 ± 1.5	ns
**AT presence** (%)	0	48	0.015 *
**LT_4_ therapy** (%)	25	37	0.448 *
**E_2_/E_1_**	3.2 ± 2.3	3.3 ± 1.9	0.584
**ACR points**	5.3 ± 0.6	7.5 ± 1.1	<0.001 *
**ACR-category**			
**4**	11	2	<0.001 *
**5**	0	25	<0.001 *

Mann–Whitney U test; *: Chi-squared test; ACR: American College of Radiology; AT: autoimmune thyroiditis: BMI: body mass index; E_2_/E_1_: elastography ratio; LT_4_: Levo-thyroxine; ns: non-significant; EU-TIRADS: European Thyroid Imaging Reporting and Data System. Three Nodules with Bethesda III and IV were excluded from the comparison.

**Table 2:** Classification table for ACR-points and malignant cytology.

Actual Group	Predicted Group		Correct (%)
	no	yes	
Bethesda II	11	0	100.0%
Bethesda V–VI	2	25	92.6%
Cases correctly classified			94.7%

ACR: American College of Radiology; Classification Criterion: ACR-points > 6.

**Table 3:** Pairwise comparison of ROC curves of our predictive models.

	ACR Points Clinical Model	ACR Points Mixed Model	Clinical Model Mixed Model
Difference between areas	0.249	0.007	0.256
Standard Error	0.084	0.007	0.082
95% Confidence Interval	0.085–0.414	−0.006–0.020	0.095–0.417
Z statistic	2.971	1.037	3.118
*p*-value	0.003	0.300	0.002

Clinical model: Multivariate logistic regression analysis considering the age, body mass index, sex, autoimmune disease, L-thyroxine therapy, nodule diameter, and elastography ratio. Mixed model: Multivariate logistic regression analysis considering the parameters of the clinical model along with the nodule’s ACR scoring points. ACR: American College of Radiology; ROC: receiver operating characteristic.

## Data Availability

Data are available from the corresponding author among reasonable requests.
